# Spin in Abstracts of Systematic Reviews and Meta-analyses of Melanoma Therapies: Cross-sectional Analysis

**DOI:** 10.2196/33996

**Published:** 2022-02-24

**Authors:** Ross Nowlin, Alexis Wirtz, David Wenger, Ryan Ottwell, Courtney Cook, Wade Arthur, Brigitte Sallee, Jarad Levin, Micah Hartwell, Drew Wright, Meghan Sealey, Lan Zhu, Matt Vassar

**Affiliations:** 1 Office of Medical Student Research Oklahoma State University Center for Health Sciences Tulsa, OK United States; 2 Department of Internal Medicine University of Oklahoma College of Community Medicine Tulsa, OK United States; 3 Department of Dermatology St. Joseph Mercy Hospital Ann Arbor, MI United States; 4 Department of Dermatology University of Oklahoma Health Sciences Center Oklahoma City, OK United States; 5 Department of Internal Medicine University of Arkansas for Medical Sciences Fayetteville, AR United States; 6 Department of Psychiatry and Behavioral Sciences Oklahoma State University Center for Health Sciences Tulsa, OK United States; 7 Samuel J. Wood Library and C.V. Starr Biomedical Information Center Weill Cornell Medical College New York, NY United States; 8 Department of Statistics Oklahoma State University Stillwater, OK United States

**Keywords:** melanoma, spin, melanoma treatment, skin conditions, skin, misinterpreting data, misinterpretation, skin cancer

## Abstract

**Background:**

Spin is defined as the misrepresentation of a study’s results, which may lead to misperceptions or misinterpretation of the findings. Spin has previously been found in randomized controlled trials and systematic reviews of acne vulgaris treatments and treatments of various nondermatological conditions.

**Objective:**

The purpose of this study was to quantify the presence of spin in abstracts of systematic reviews and meta-analyses of melanoma therapies and identify any related secondary characteristics of these articles.

**Methods:**

We used a cross-sectional approach on June 2, 2020, to search the MEDLINE and Embase databases from their inception. To meet inclusion criteria, a study was required to be a systematic review or meta-analysis pertaining to the treatment of melanoma in human subjects, and reported in English. We used the PRISMA (Preferred Reporting Items for Systematic Reviews and Meta-Analyses) definition of systematic reviews and meta-analyses. Data were extracted in a masked, duplicate fashion. We conducted a powered bivariate linear regression and calculated odds ratios for each study characteristic.

**Results:**

A total of 200 systematic reviews met the inclusion criteria. We identified spin in 38% (n=76) of the abstracts. The most common type of spin found was type 3 (selective reporting of or overemphasis on efficacy outcomes or analysis favoring the beneficial effect of the experimental intervention), occurring 40 times; the least common was type 2 (title claims or suggests a beneficial effect of the experimental intervention not supported by the findings), which was not present in any included abstracts. We found that abstracts pertaining to pharmacologic interventions were 3.84 times more likely to contain spin. The likelihood of an article containing spin has decreased annually (adjusted odds ratio 0.91, 95% CI 0.84-0.99). No significant correlation between funding source or other study characteristics and the presence of spin was identified.

**Conclusions:**

We have found that spin is fairly common in the abstracts of systematic reviews of melanoma treatments, but the prevalence of spin in these abstracts has been declining from 1992-2020.

## Introduction

Skin cancer is the most common form of cancer in the United States, with more than 9500 new diagnoses each day [1]. Among skin cancer types, melanoma remains the most deadly, responsible for an estimated 6850 deaths in 2020 [2]. Furthermore, the incidence of melanoma is projected to rise by 2% in 2020, continuing a trend that has existed for more than 6 decades [2,3]. Although the standard treatment for melanoma is surgical excision, new therapies have recently emerged, including targeted therapies (such as BRAF and MEK inhibitors) and immunotherapies (such as anti-PD1 and anti–CTLA-4 antibodies), which have contributed to a recent decrease in mortality rates [2,4]. An increase in the volume of published research, in tandem with an increased number of available effective therapies, has resulted in a substantial number of studies for dermatologists to consider when recommending melanoma therapies to their patients. For this reason, systematic reviews have become an essential tool for clinicians, making accurate reporting of the results in both abstracts and manuscripts an integral component of scientific writing.

The term *spin* has been defined as “specific reporting that could distort the interpretation of results and mislead readers” [5,6]. Although abstracts are historically viewed as compressed versions of a full manuscript, scientists may highlight specific findings in the abstract to make the study’s results appear more compelling [6] and engage more readers [7]. Clinicians endeavoring to maintain an up-to-date evidence-based practice often rely on an abstract alone to formulate a clinical opinion [8-10]. One study found that clinicians were 2.4 times more likely to read an abstract than an entire article [11]. Therefore, it is not an unfair assumption that a study abstract may directly influence a dermatologist's approach to melanoma management, especially considering the breadth of new and emerging therapies and combination regimens.

Notwithstanding clinicians’ reliance on systematic reviews in everyday decision-making, it has been demonstrated that reporting in the abstracts of systematic reviews is frequently flawed [12-15]. The presence of spin has been exhibited in abstracts of randomized controlled trials (RCTs) in a multitude of specialties, including psychiatry [16], anesthesiology [17], oncology [18], and emergency medicine [19], revealing significant issues of transparency in the reporting of results in published abstracts. Ottwell et al [20] recently identified spin in almost one-third of systematic reviews and meta-analyses of acne vulgaris therapies. In this study, we aimed to evaluate the presence of spin in abstracts of systematic reviews and meta-analyses focused on melanoma treatment. Additionally, we discuss the clinical repercussions if clinicians are presented with misleading information and provide recommendations to reduce spin and improve overall reporting in systematic reviews and meta-analyses.

## Methods

### Oversight, Transparency, Reproducibility, and Reporting

As no humans were involved in this study, it did not meet the regulatory definition of human subject research per the US Code of Federal Regulations and was not subject to institutional review board oversight. The associated protocol, extraction forms, data analysis scripts, and other study artifacts have been uploaded to Open Science Framework to ensure transparency and reproducibility [21]. To further ensure the reproducibility of our analyses, the data were reanalyzed in a masked fashion by a third-party statistician. This study was conducted concurrently with similar studies evaluating the presence of spin in systematic reviews in other fields of medicine. These studies adhered to a common methodology that has been described elsewhere [20]. The relevant reporting guidelines were incorporated in the drafting of this manuscript, specifically PRISMA (Preferred Reporting Items for Systematic Reviews and Meta-Analyses) [22] and Murad and Wang's [23] guidelines for meta-epidemiological studies.

### Search Strategy

A study team member (DW), a systematic review librarian, constructed search strategies for the MEDLINE (Ovid) and Embase (Ovid) databases and used them to locate systematic reviews and meta-analyses of treatment modalities for melanoma (Textbox 1).

Both databases were searched from their inception. DW conducted these searches on June 2, 2020; the retrieved records were uploaded to Rayyan, a systematic review screening platform [24]. After duplicates were removed, two authors (RN and AW) independently screened the titles and abstracts of the remaining records to determine eligibility.

**Search queries.**

**Ovid MEDLINE**

1. exp Melanoma/

2. (melanoma* or (pigment* adj1 cancer*) or melanocarcinoma* or nevocarcinoma*).mp.

3. 1 or 2

4. exp Therapeutics/

5. (treat* or therap* or help* or interven*).mp.

6. 4 or 5

7. 3 and 6

8. exp Melanoma/dh, dt, th [Diet Therapy, Drug Therapy, Therapy]

9. 7 or 8

10. exp ”Systematic Review“/

11. exp Meta-Analysis/

12. (”systematic review“ or ”meta-analysis“ or (systematic* adj1 review*)).ti,ab.

13. 10 or 11 or 12

14. 9 and 13

**Ovid Embase**

1. exp melanoma/

2. (melanoma* or (pigment* adj1 cancer*) or melanocarcinoma* or nevocarcinoma*).mp.

3. 1 or 2

4. exp therapy/

5. (treat* or therap* or help* or interven*).mp.

6. 4 or 5

7. 3 and 6

8. exp melanoma/dm, dt, th [Disease Management, Drug Therapy, Therapy]

9. 7 or 8

10. exp ”systematic review“/

11. exp meta analysis/

12. (”systematic review“ or ”meta-analysis“ or (systematic* adj1 review*)).ti,ab.

13. 10 or 11 or 12

14. 9 and 13

### Eligibility Criteria

Studies were required to meet the following inclusion criteria: (1) a systematic review with or without a meta-analysis; (2) focused on the treatment of melanoma; (3) conducted on human subjects only; and (4) available in English. We used the PRISMA definition of systematic reviews and meta-analyses [25]. Studies that met these criteria were uploaded to Stata 16.1 (StataCorp LLC) for randomization. Data were then extracted from the first 200 systematic reviews.

### Training

Before title and abstract screening commenced, authors RN and AW completed an online training course on systematic reviews and meta-analyses by Li and Dickersin [26]. They then completed 2 days of online and in-person training on the definition and interpretation of the 9 most severe types of spin in systematic review abstracts [27]*.* Finally, they were trained in A MeaSurement Tool to Assess systematic Reviews (AMSTAR-2), a frequently used 16-item instrument for measuring the methodological quality of systematic reviews and meta-analyses [28]. A detailed outline of the training regimen can be found in our study protocol.

### Data Extraction

Data were extracted in a masked, duplicate fashion using a pilot-tested Google form. Abstracts of the included systematic reviews were thoroughly examined for the presence of the 9 most severe types of spin. The 9 spin types, defined by Yavchitz et al [27], are as follows: (1) conclusion contains recommendations for clinical practice not supported by the findings, (2) title claims or suggests a beneficial effect of the experimental intervention not supported by the findings, (3) selective reporting of or overemphasis on efficacy outcomes or analysis favoring the beneficial effect of the experimental intervention, (4) conclusion claims safety based on non–statistically significant results with a wide confidence interval, (5) conclusion claims the beneficial effect of the experimental treatment despite high risk of bias in primary studies, (6) selective reporting of or overemphasis on harm outcomes or analysis favoring the safety of the experimental intervention, (7) conclusion extrapolates the review’s findings to a different intervention (ie, claiming efficacy of one specific intervention although the review covers a class of several interventions), (8) conclusion extrapolates the review’s findings from a surrogate marker or a specific outcome to the global improvement of the disease, and (9) conclusion claims the beneficial effect of the experimental treatment despite reporting bias.

The methodological quality of each study was rated as high, moderate, low, or critically low using the AMSTAR-2 scale [28]. In previous studies, the interrater reliability of AMSTAR-2 scores has been moderate to high, with high construct validity coefficients associated with both the original AMSTAR instrument (*r*=0.91) and the Risk of Bias in Systematic Reviews instrument (*r*=0.8429) [29].

The study characteristics extracted from each systematic review and meta-analysis were as follows: (1) type of intervention (surgery, pharmacologic, nonpharmacologic, combination, other); (2) date the review was received by the journal; (3) funding sources (hospital, industry, private, public, a combination of sources including industry, a combination of sources excluding industry, none, not mentioned, other); (4) whether the review discussed compliance with PRISMA or PRISMA for Abstracts [30]; (5) whether the journal required compliance with PRISMA; (6) the journal’s word limit for abstracts, if any; and (7) the journal's 5-year impact factor. Once data extraction was complete, authors RN and AW were unmasked. If possible, discrepancies were resolved by consensus. Author RO adjudicated if consensus could not be achieved.

### Statistical Analysis

The overall frequency of spin and its subtypes was characterized using descriptive statistics. We then used unadjusted logistic regression models to determine the binary associations of impact of extracted study characteristics on the presence of spin in the abstracts of systematic reviews and meta-analysis. We then constructed a multivariable logistic regression model to determine the influence of these variables, controlling for each, on the presence of spin. In our protocol, we prespecified the possibility of a binary logistic regression and calculated a power analysis before the start of this study to determine required sample size using GPower (version 3.1.9.7). A previous investigation of spin in abstracts of systematic reviews and meta-analyses focused on acne vulgaris suggested that spin was present in 31% of abstracts. We therefore based our power analysis on the following assumptions and parameters: (1) twenty percent of PRISMA-compliant systematic reviews and 40% of non–PRISMA-compliant systematic reviews contain spin; (2) a type I error rate of .05 (2-tailed); (3) power of .80; and (4) multiple coefficients of determination of 0.10. We thus concluded that 185 systematic reviews would be needed. These analytic decisions are documented in our protocol. We used Stata 16.1 for all analyses.

## Results

### General Characteristics

**Figure 1:**
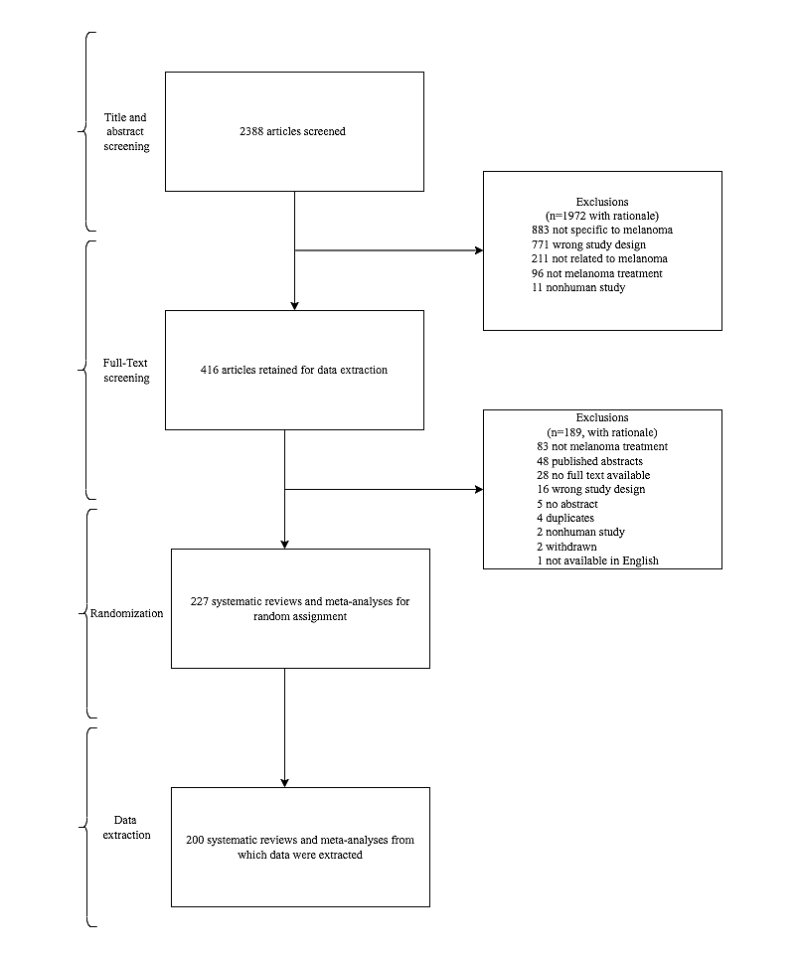
PRISMA (Preferred Reporting Items for Systematic Reviews and Meta-Analyses) flow diagram demonstrating all steps of article screening with rationale provided for excluded articles.

Our initial search returned 3106 unique articles, of which 718 were removed as duplicates. An additional 1972 articles were excluded during title and abstract screening. Full-text screening resulted in the exclusion of 189 articles. Thus, 227 systematic reviews met inclusion criteria and underwent random assignment, following which data were extracted from 200. Our screening (with rationale for exclusions) and randomization process is illustrated in Figure 1.

The most common intervention type was pharmacologic (115/200, 57.5%), followed by surgical interventions (38/200, 19%). The date range during which included systematic reviews were received by their publishing journal spanned from 1992 to 2020 (Table 1).

**Table 1:** General characteristics of systematic reviews and meta-analyses.

Characteristics		Articles (N=200)			Odds ratio (95% CI)	
		Total	Abstract contains spin	Unadjusted		Adjusted
**Intervention type, n (%)**						
	Mixed	32 (16)	6 (3)	1 (Reference)		1 (Reference)
	Nonpharmacologic	15 (7.5)	7 (3.5)	3.79 (0.98-14.60)		4.69 (0.73-30.10)
	Pharmacologic	115 (57.5)	54 (27)	3.84 (1.46-10.02*)*		2.60 (0.64-10.61)
	Surgery	38 (19)	9 (4.5)	1.34 (0.42-4.29)		1.25 (0.24-6.35)
**Study mentions adherence to PRISMA,^a^ n (%)**						
	No	119 (59.5)	41 (20.5)	1 (Reference)		1 (Reference)
	Yes	81 (40.5)	35 (17.5)	1.45 (0.81-2.58)		1.24 (0.49-3.13)
**Publishing journal recommends adherence to PRISMA, n (%)**						
	No	98 (49)	40 (20)	1 (Reference)		1 (Reference)
	Yes	102 (51)	36 (18)	0.79 (0.44-1.40)		0.55 (0.25-1.24)
**Funding source, n (%)**						
	Not funded	46 (23)	15 (7.5)	1 (Reference)		1 (Reference)
	Industry	27 (13.5)	14 (7)	2.23 (0.84-5.90)		2.08 (0.58-7.41)
	Not mentioned	86 (43)	29 (14.5)	1.05 (0.49-2.25)		0.54 (0.18-1.61)
	Private	24 (12)	8 (4)	1.03 (0.36-2.95)		0.74 (0.20-2.79)
	Public	17 (8.5)	10 (5)	2.95 (0.94-9.29)		1.50 (0.35-6.44)
**AMSTAR-2^b^ rating, n (%)**						
	High	17 (8.5)	6 (3)	1 (Reference)		1 (Reference)
	Moderate	47 (23.5)	27 (13.5)	2.48 (0.78-7.82)		1.83 (0.47-7.19)
	Low	19 (9.5)	11 (5.5)	2.52 (0.65-9.71)		3.05 (0.60-15.48)
	Critically low	117 (58.5)	32 (16)	0.69 (0.24-2.02)		0.45 (0.11-1.86)
5-year impact factor, mean (SD)		6.02 (6.57)	6.84 (7.36)	1.03 (0.98-1.08)		1.04 (0.98-1.10)
Abstract word limit, mean (SD)		281 (125.35)	276 (115.84)	1.00 (1.00-1.00)		1.00 (0.99-1.00)
Publication year (1992-2020)		N/A^c^	N/A	0.99 (0.93-1.04)		0.91 (0.84-0.99)

^a^PRISMA: Preferred Reporting Items for Systematic Reviews and Meta-Analyses.

^b^AMSTAR-2: A MeaSurement Tool to Assess systematic Reviews.

^c^N/A: not applicable.

Of 200 studies, 68 (34%) were funded, with the most common funding source being industry (27/200, 13.5%), while 46 studies were not funded (46/200, 23%) and 86 did not mention a funding source (86/200, 43%). Most studies did not mention adherence to PRISMA (119/200, 59.6%) and a total of 102 studies (51%) were published in journals whose submission guidelines recommend PRISMA adherence. The average word limit for abstracts was 281 (SD 125.35). The average 5-year impact factor for our sample was 6.02 (SD 6.57).

### Spin in Abstracts of Systematic Reviews and Meta-analyses

Among the 200 studies in our sample, we found spin in 76 (38%) of the abstracts. We frequently found more than 1 type of spin in an abstract; thus, 117 instances of spin were identified. Spin type 3—selective reporting of or overemphasis on efficacy outcomes or analysis favoring the beneficial effect of the experimental intervention—was the most common, occurring in 40 abstracts (20%; Table 2).

**Table 2:** Spin types and frequencies (%) in abstracts (N=200).

Nine most severe types of spin [27]	Abstracts containing spin, n (%)
1. Conclusion contains recommendations for clinical practice not supported by the findings.	4 (2)
2. Title claims or suggests a beneficial effect of the experimental intervention not supported by the findings.	0 (0)
3. Selective reporting of or overemphasis on efficacy outcomes or analysis favoring the beneficial effect of the experimental intervention.	40 (20)
4. Conclusion claims safety based on nonstatistically significant results with a wide confidence interval.	3 (7.1)^a^
5. Conclusion claims the beneficial effect of the experimental treatment despite high risk of bias in primary studies.	16 (8)
6. Selective reporting of or overemphasis on harm outcomes or analysis favoring the safety of the experimental intervention.	27 (13.5)
7. Conclusion extrapolates the review’s findings to a different intervention (ie, claiming efficacy of one specific intervention although the review covers a class of several interventions).	4 (2)
8. Conclusion extrapolates the review’s findings from a surrogate marker or a specific outcome to the global improvement of the disease.	13 (6.5)
9. Conclusion claims the beneficial effect of the experimental treatment despite reporting bias.	10 (5)

^a^A total of 158 abstract conclusions did not mention safety, thus n=42.

The most severe type of spin, type 1—conclusion contains recommendations for clinical practice not supported by the findings—occurred in 4 abstracts (2%). Because 158 studies did not mention safety outcomes or safety measures in their conclusions, only 42 abstracts could be assessed for spin type 4 (3/42, 7.1%). No abstracts contained spin type 2.

**Figure 2:**
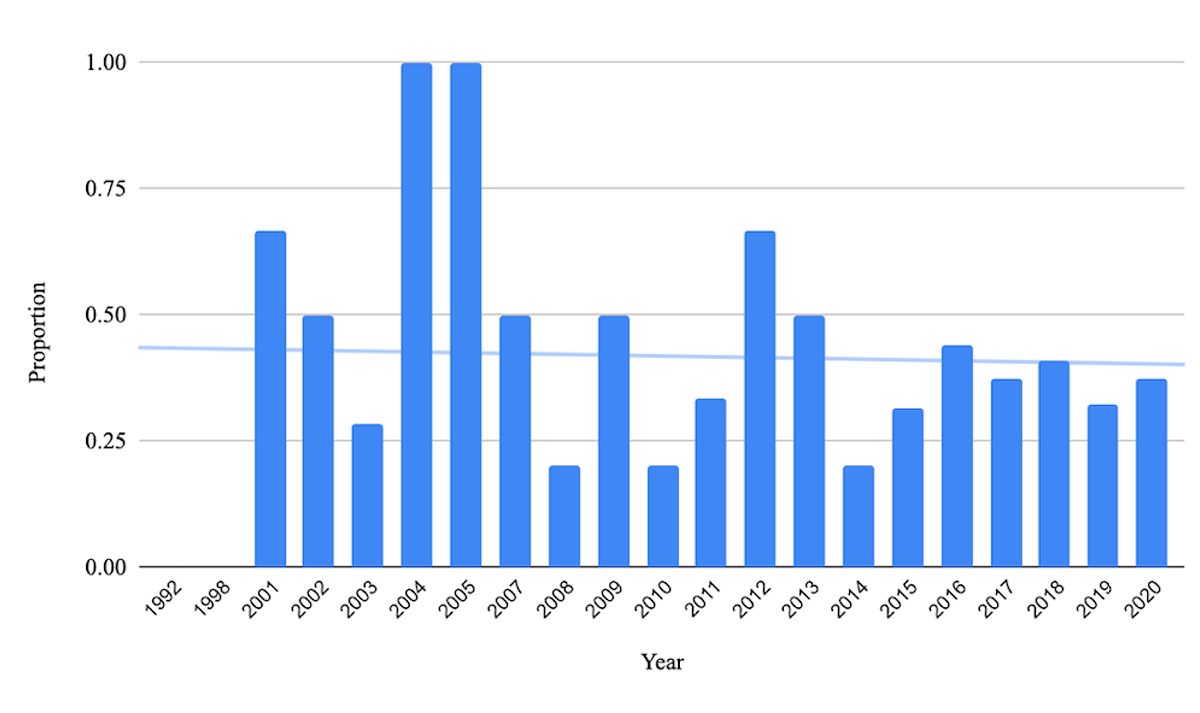
The proportion of systematic reviews containing spin in the abstract from 1992-2020.

From the bivariate logistic regression, the odds were 384% higher for a systematic review covering pharmacologic interventions to contain spin compared with the reference group (odds ratio [OR] 3.84, 95% CI 1.46-10.2). After adjustment for possible covariates, this association between spin and pharmacologic interventions did not remain statistically significant (OR 2.60, 95% CI 0.64-10.61). We found that the likelihood of an article containing spin has decreased annually (adjusted OR 0.91, 95% CI 0.84-0.99; Table 1). Figure 2 illustrates the proportion and overall downward trend of spin prevalence in abstracts of systematic reviews focused on melanoma therapies from 1992 to 2020. We found no other association between the presence of spin and other study characteristics.

### AMSTAR-2 Ratings

A total of 58.5% (117/200) of systematic reviews in our sample received a methodological quality rating of “critically low” on the AMSTAR-2 scale, 9.5% (19/200) were rated “low” quality, 23.5% (47/200) “moderate” quality, and 8.5% (17/200) “high” quality. The presence of spin was not significantly associated with a study’s AMSTAR-2 rating. All AMSTAR-2 items and frequency of responses are found in Table 3.

**Table 3:** AMSTAR-2^a^ items and frequency of responses (N=200).

AMSTAR-2 item	Response, n (%)		
	Yes	No	Partial yes
1. Did the research questions and inclusion criteria for the review include the elements of PICO (patient/population, intervention, comparison, and outcomes)?	200 (100)	0 (0)	0 (0)
2. Did the report of the review contain an explicit statement that the review methods were established prior to the conduct of the review and did the report justify any significant deviations from the protocol?	66 (33)	75 (37.5)	59 (29.5)
3. Did the review authors explain their selection of the study designs for inclusion in the review?	103 (51.5)	97 (48.5)	0 (0)
4. Did the review authors use a comprehensive literature search strategy?	37 (18.5)	54 (27)	109 (54.5)
5. Did the review authors perform study selection in duplicate?	121 60.5)	79 (39.5)	0 (0)
6. Did the review authors perform data extraction in duplicate?	126 (63)	74 (37)	0 (0)
7. Did the review authors provide a list of excluded studies and justify the exclusions?	15 (7.5)	65 (32.5)	120 (60)
8. Did the review authors describe the included studies in adequate detail?	46 (23)	23 (11.5)	131 (65.5)
9. Did the review authors use a satisfactory technique for assessing the risk of bias in individual studies that were included in the review?	51 (28.5)^b^	104 (58.1)^b^	24 (13.4)^b^
10. Did the review authors report on the sources of funding for the studies included in the review?	20 (10)	180 (90)	0 (0)
11. If meta-analysis was performed, did the review authors use appropriate methods for statistical combination of results?	95 (93.1)^c^	7 (6.9)^c^	0 (0)^c^
12. If meta-analysis was performed, did the review authors assess the potential impact of risk of bias in individual studies on the results of the meta-analysis or other evidence synthesis?	62 (60.7)^c^	40 (39.2)^c^	0 (0)^c^
13. Did the review authors account for risk of bias in primary studies when interpreting/discussing the results of the review?	74 (37)	126 (63)	0 (0)
14. Did the review authors provide a satisfactory explanation for, and discussion of, any heterogeneity observed in the results of the review?	121 (60.5)	79 (39.5)	0 (0)
15. If they performed quantitative synthesis, did the review authors carry out an adequate investigation of publication bias (small study bias) and discuss its likely impact on the results of the review?	53 (52)^c^	49 (48)^c^	0 (0)^c^
16. Did the review authors report any potential sources of conflict of interest, including any funding they received for conducting the review?	163 (81.5)	37 (18.5)	0 (0)

^a^AMSTAR-2: A MeaSurement Tool to Assess systematic Reviews.

^b^A total of 21 articles included only nonrandomized studies of interventions and were not included in the table, thus N=179.

^c^A total of 98 articles did not perform a meta-analysis, thus N=102.

## Discussion

### Primary Findings

Our study suggests that approximately 1 in 3 systematic reviews or meta-analyses focused on melanoma treatment modalities contain spin in their abstract. The most common type of spin identified in our sample was type 3—selective reporting of or overemphasis on efficacy outcomes or analysis favoring the beneficial effect of the experimental intervention. An example of such selective reporting occurred in a study by Verma et al [31], which reviewed systemic adjuvant therapies for patients at high risk for recurrent melanoma. The primary outcomes included overall survival, recurrence-free survival, adverse effects, and quality of life; however, the abstract failed to mention 3 of the 4 outcomes (recurrence-free survival, adverse effects, and quality of life). The selective omittance of primary outcomes in an abstract has the potential to allow readers to make assumptions regarding omitted outcomes based on the positive or negative nature of the outcomes that are reported. This finding is concerning as clinicians often use abstracts to guide clinical decisions. Because omitting primary outcomes may affect patient care [9,32,33], it is imperative that abstracts contain full information about both efficacy and adverse events.

An interesting finding was the frequency with which spin type 6 (selective reporting of or overemphasis on harm outcomes or analysis favoring the safety of the experimental intervention) occurred concurrently with spin type 3 (30.7%). For example, Dafni et al [34] reported overall survival and toxicities as 2 of their secondary outcomes but selectively did not report these findings alongside the other stated secondary outcomes. This example of the concurrent occurrence of spin types 3 and 6 demonstrates how selective reporting of efficacy and harm outcomes could distort a reader's interpretation of the full benefits and risks of an experimental regimen. This is especially important as we found that systematic reviews focused on pharmacologic interventions, which are often associated with higher toxicity profiles [35,36], had increased odds of containing spin. Thus, it is essential that clinicians recognize spin and its potential influence on therapeutic recommendations.

To incorporate our findings into the existing body of literature on spin, we must compare our results with previous evaluations of spin in RCTs and observational studies. Our team’s previous investigations found spin in abstracts at rates ranging from 37% in oncology RCTs [18] to 70% in otolaryngology RCTs [37]. More recently, studies have shown that spin frequently occurs in abstracts of systematic reviews [38-48]. As previously mentioned, Ottwell et al [20] identified spin in 31% of the included abstracts of systematic reviews and meta-analyses on acne vulgaris therapies, a finding similar to ours. Although the presence of any amount of spin is relevant as it may mislead readers, it should be noted that our findings suggest that abstracts of systematic reviews focused on melanoma treatment appear to contain equal or fewer amounts of spin than their counterparts in other fields of medicine and may be improving with time.

In 2013, PRISMA released its extension for abstracts [30], an initiative to improve the quality of reporting in abstracts. However, findings are mixed on whether the release of PRISMA for Abstracts has improved the quality of abstract reporting. Interestingly, one consistent finding across these studies [49,50] is that authors do not report all 12 PRISMA for Abstracts items. A study by O’Donohoe et al [14] found that systematic reviews published in journals with higher abstract word limits had significantly higher PRISMA for Abstracts reporting scores. This finding seems logical, as higher word limits would allow all 12 items to be reported and permit the reporting of all outcomes, thus reducing the occurrence of selective-reporting spin. Although our study did not show that higher abstract word limits reduced spin, greater freedom for authors in regard to word limits seems justified as systematic reviews are considered the “gold standard” of scientific evidence and their abstracts have been shown to have a role in clinical decisions [9,32].

### Strengths and Limitations

Our study was conducted in a fashion that maximized reproducibility and transparency. This was achieved by publishing our protocol (before the investigation’s start date), all data, and training modules to the Open Science Framework. Additional statistical reproducibility was achieved by having all data analyses confirmed by an independent group. A final strength is that data were extracted in a duplicated and masked fashion, which the Cochrane Collaboration considers to be the gold standard [51].

Regarding limitations, the assessment of spin is inherently subjective. To reduce subjectivity, the investigators completed several days of online and in-person training in strictly defining spin and identifying its presence. Additionally, because we searched only 2 databases (MEDLINE and Embase), some relevant studies may have been missed. Specific study characteristics had inherent limitations. For example, some studies were published before the release of PRISMA. It is unclear when journals began recommending PRISMA guidelines as previous author guidelines were not available. In addition, owing to the wide date range of published studies, we used 5-year impact factors to account for variations, which may not accurately reflect past journal impact factors. Lastly, the tool we used to appraise systematic reviews, the AMSTAR-2, was developed and published in 2017; thus, using it to rate systematic reviews published before 2017 may have resulted in lower scores.

### Conclusion

In summary, we found spin in 38% of abstracts of systematic reviews and meta-analyses pertaining to melanoma treatment. Our results indicate that the incidence of spin in abstracts of systematic reviews focused on melanoma therapies is on par with or less than the incidence reported by investigations in other medical fields. Additionally, our results show that spin in abstracts of systematic reviews focused on melanoma therapies is decreasing. The fields of dermatology and oncology therefore have the opportunity to be leaders in reducing abstract spin prevalence and improving the quality of reporting in abstracts of systematic reviews focused on melanoma treatment.

## Abbreviations

AMSTAR-2: A MeaSurement Tool to Assess systematic Reviews
PRISMA: Preferred Reporting Items for Systematic Reviews and Meta-Analyses
RCT: randomized controlled trial
